# Angiogenic miRNAs, the angiopoietin axis and related TIE2-expressing monocytes affect outcomes in cholangiocarcinoma

**DOI:** 10.18632/oncotarget.25699

**Published:** 2018-07-06

**Authors:** Georgi Atanasov, Corinna Dietel, Linda Feldbrügge, Christian Benzing, Felix Krenzien, Andreas Brandl, Shadi Katou, Katrin Schierle, Simon C. Robson, Katrin Splith, Georg Wiltberger, Anja Reutzel-Selke, Sven Jonas, Andreas Pascher, Marcus Bahra, Johann Pratschke, Moritz Schmelzle

**Affiliations:** ^1^ Department of Surgery, Charité–Universitätsmedizin Berlin, Berlin, Germany; ^2^ Berlin Institute of Health, Berlin, Germany; ^3^ Department of Visceral, Transplantation, Thoracic and Vascular Surgery, University Hospital Leipzig, Leipzig, Germany; ^4^ Institute of Pathology, University Hospital Leipzig, Leipzig, Germany; ^5^ The Transplant Institute and Division of Gastroenterology, Beth Israel Deaconess Medical Center, Harvard University, Boston, MA, USA; ^6^ Department of General, Visceral and Transplantation Surgery, University Hospital of RWTH Aachen, Aachen, Germany; ^7^ Department of General and Visceral Surgery, 310Klinik Nürnberg, Nürnberg, Germany

**Keywords:** cholangiocarcinoma, TIE2-expressing monocytes, microRNAs, angiopoietins, miR-126

## Abstract

**Background:**

Tumour angiogenesis is modulated on both an epigenetic and protein level and has potential implications for immune cell responses. However, the importance of related angiogenic biomarkers in cholangiocarcinoma (CCA) is unknown. This study assessed human CCA samples for the expression of angiogenesis-associated microRNAs, angiopoietins (Angs) and monocytes expressing the Ang-receptor, TIE2, with regards to prognostic significance after liver resection.

**Methods:**

Angiogenic miRNAs were analysed in frozen samples of intrahepatic CCA (iCC; n = 43) and hilar CCA (HC; n = 45). Ang-1 and Ang-2, as well as TIE2-expressing monocytes (TEMs), were detected in paraffin-embedded iCC sections (n = 88). MiRNA expression and the abundance of TEMs and Angs were correlated with clinicopathological characteristics and survival.

**Results:**

MiR-126 was downregulated in 76.7% of all CCA samples, with high relative expression associated with smaller tumours and reduced lymph node metastasis. High Ang-1 expression was associated with less lymphangiosis carcinomatosa and better histological grading (all p < 0.05). The absence of TEMs in iCC correlated with elevated CA19-9 levels. High relative miR-126 and low miR-128 levels were associated with improved survival in iCC and HC, respectively (all p < 0.05). High miR-126, low miR-128 and TEMs were independent prognostic factors for recurrence-free and overall survival (all p < 0.05).

**Conclusions:**

These results suggest that angiogenic miRNAs, Angs and TEMs are of prognostic value in CCA. In addition to the possible functional links between angiogenic miRNA expression profiles, Angs and immune-cell responses by TEMs, these data have clinical implications as novel diagnostic tools.

## INTRODUCTION

MicroRNAs (miRNAs) are a large family of endogenous small noncoding RNAs (19–24 nucleotides) that play a crucial regulatory role by binding to the 3′ untranslated regions of target mRNAs and blocking translation or initiating mRNA degradation [1, 2]. Accumulating evidence suggests that miRNAs regulate tumour proliferation, invasion, apoptosis and therapy resistance. MiRNAs can act as oncogenes or tumour suppressors depending on the target mRNAs, and they qualify as promising biomarkers [3–5]. Moreover, miRNAs are crucially involved in the processes of vascular development and remodelling and angiopoietin axis-dependent tumour angiogenesis [6–8].

Recent research revealed that mature vessels express high levels of miR-126, which promotes the remodelling and stabilisation effects of angiopoietin-1 (Ang-1) [9]. Another angiogenic miRNA, miR-128, which is related to vascular endothelial growth factor (VEGF)-C translation and protein expression, is differentially expressed in several types of human cancer and impacts cancer cell growth and invasion. However, the published data is inconsistent with regards to the possible beneficial or negative impacts of these angiogenic miRNAs in the promotion of human malignancies [10–12]. In poorly vascularised human cholangiocarcinoma (CCA), the role of miR-128, miR-126 and other angiogenesis-related miRNAs remains unknown.

Ang family members and VEGF are potent growth factors and important modulators of tumour-related angiogenesis; however, they could also represent functional antagonists [13]. VEGF is required for the formation of the initial vascular plexus in the early phases of development, whilst Ang-1 is necessary for subsequent vascular remodelling into mature blood vessels [14]. In addition, VEGF can cause vascular instability by promoting permeability and tissue oedema, whilst Ang-1 stabilises existing vessels and decreases vascular leakage [15, 16]. Recent studies highlighted the additional beneficial roles of Ang-1 in cancer models [17–19]. In contrast, the overexpression of angiopoietin-2 (Ang-2) was associated with an advanced disease state and poor prognosis in different solid malignancies, thus suggesting differential roles of Ang-1 and Ang-2 in tumour biology [20–22].

Tie2-expressing monocytes (TEMs) were recently shown to exert profound proangiogenic activities and constitute preeminent immunological compounds that were distinct from tumour-associated macrophages (TAMs) [23–24]. The tyrosine kinase with Ig and EGF homology domains 2 (TIE2) is a receptor binding to all known Angs. TEMs express functional TIE2 receptor and respond directly to Angs activity. These immune cells foster tumour angiogenesis and progression in various models of animal and human cancer [25–27]. However, as is already apparent for TAMs, data also imply that TEMs have a beneficial impact in human malignancies [28]. These effects possibly depend on the vascularisation grade of solid tumours and the associated molecular angiogenic pathways.

Angiogenic miRNAs, Angs and TEMs were suggested to have clinical significance in human malignancies where the interplay between tumour immunology and angiogenesis plays a central role in tumour progression [23]. TEMs express angiogenic factors such as basic fibroblast growth factor and VEGF and are causally involved in hepatocarcinogenesis [29]. In hypoxic conditions, activated endothelial cells secrete Angs and recruit TEMs to the tumour site, thus delineating the importance of the Ang–TIE2 axis and TEMs in the context of vascular-rich tumours, such as hepatocellular carcinoma (HCC) [30, 31]. However, the significance of these angiogenic immune markers in poorly-vascularised intrahepatic CCA (iCC) is unknown. It was previously shown that Ang-1 density was associated with beneficial tumour characteristics in hilar CCA (HC) and that corresponding TEMs positively affected patient survival and prognosis [28]. The current work hypothesised that miR-126 signalling upregulates Ang-1-dependent direct beneficial effects in hepatocarcinogenesis and indirect effects by fostering the homing of TEMs in tumours, which in turn exert a significant impact on CCA progression. Therefore the aim of the study was to evaluate the significance of angiogenic miRNAs, Angs and related TEMs at the epigenetic, protein and cellular level. The study also aimed to determine their association with tumour growth, metastasis, recurrence and clinical prognosis in human CCA.

## RESULTS

### MiRNAs regulating (lymph) angiogenic factors are differentially expressed in CCA

**Figure 1:**
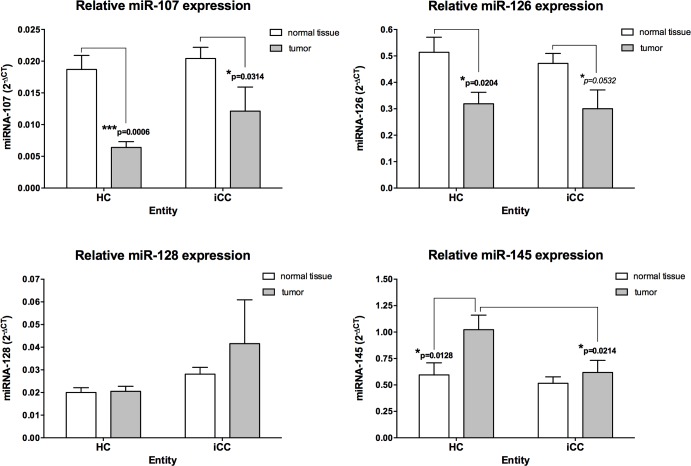
Angiogenic micro-RNAs in hilar (HC; n = 45) and intrahepatic (iCC; n = 43) cholangiocarcinoma The relative expression was calculated using the comparative ΔΔC_T_ method, and the values were expressed as 2^−ΔCT^ compared to normal liver tissue. MiR-107 and miR-126 showed a strong downregulation in HC and iCC, compared to adjacent noncancerous liver tissue. MiR-145 in HC revealed a strong upregulation, when compared with normal tissue or iCC (all p<0.05).

The relative expression levels of several miRNAs thought to regulate vascular growth factors involved in Ang–TIE2 signalling were initially assessed in CCA samples [9, 32–36]. MiR-126, miR-145, miR-128 and miR-107 were detected in all CCA samples. We identified miR-145 with highest median relative level in CCA when compared with adjacent noncancerous liver tissue (Figure 1). Of note, MiR-145 in HC revealed a strong up-regulation, when compared with normal tissue or iCC (p = 0.0128 and p = 0.0214, respectively). On the contrary, relative miR-107 and miR-126 expression levels in HC and iCC revealed a significant down-regulation, in comparison with normal tissue (all p ≤ 0.05; Figure 1).

### MiRNA-126 is downregulated in CCA and associated with lymph node involvement and tumour growth

MiR-126 was proposed as a potent tumour suppressor that regulates Ang-1 signalling [9]. The relative expression of miR-126 was strongly downregulated in 32 HC subjects (71.1%) and 33 iCC subjects (76.7%). A strong upregulation of miR-126 was apparent in only nine (20.0%) HC subjects and six (13.9%) iCC subjects.

In HC, high miR-126 relative expression was associated with a smaller tumour mass (largest tumour diameter < 50 mm) (Table 1). All 27 (100%) cases with high relative miR-126 expression in the tumour exhibited a smaller tumour size. Conversely, only seven (38.8%) of the 18 patients with low relative miR-126 expression in the tumour exhibited a smaller tumour size (p = 0.0001). In iCC, high relative miR-126 expression was associated with reduced lymph node metastasis (p = 0.013). It is noteworthy that 13 (92.9%) of the 14 iCC patients with high relative miR-126 expression in the tumour showed no lymph node involvement.

**Table 1:** Correlation of microRNA-126 relative expression in tumor with clinicopathological characteristics of patient with cholangiocarcinoma

Clinicopathological analysis in hilar cholangiocarcinoma			
Variable	miR-126 low	miR-126 high	P
No. of patients	18	27	
Gender			0.895
Female	8 (44.4%)	13 (46.4%)	
Male	10 (55.6%)	14 (53.6%)	
Patient age, years			0.170
≤ 60	10 (55.6%)	20 (75.0%)	
> 60	8 (44.4%)	7 (25.0%)	
Tumor size			0.0001
≤ 50 mm	8 (44.4%)	27 (100%)	
> 50 mm	10 (55.6%)	0 (0%)	
Pathologic N stage			0.542
Positive	7 (38.8%)	14 (51.9%)	
Negative	11 (61.2%)	13 (48.1%)	
Histologic differentiation			0.798
Well	4 (22.2%)	4 (14.8%)	
Moderate/poor	13 (77.8%)	23 (85.2%)	
**Clinicopathological analysis in intrahepatic cholangiocarcinoma**			
**Variable**	**miR-126 low**	**miR-126 high**	**p**
No. of patients	29	14	
Gender			0.676
Female	15 (55.6%)	8 (57.1%)	
Male	14 (44.4%)	6 (42.9%)	
Patient age, years			0.524
≤ 60	12 (41.4%)	4 (28.6%)	
> 60	17 (58.6%)	10 (71.4%)	
Tumor size			0.967
≤ 50 mm	9 (31.0%)	4 (28.6%)	
> 50 mm	20 (69.0%)	10 (71.4%)	
Pathologic N stage			0.013
Positive	14 (47.4%)	1 (7.1%)	
Negative	15 (52.6%)	13 (92.9%)	
Histologic differentiation			0.529
Well	20 (69.0%)	9 (64.3%)	
Moderate/poor	9 (31.0%)	5 (35.7%)	

### Angiopoietin expression is associated with lymphangiosis carcinomatosa and histological grading in iCC

Ang-1 was previously shown to be associated with beneficial tumour characteristics in HC [31]. In the current work, Ang-1 distribution revealed a uniform pattern in all tumour areas, including the tumour-infiltrating front. The adjacent liver parenchyma also exhibited Ang-1 expression; however, the pattern was less evident than in the tumour tissue (data not shown).

In iCC, the high density of Ang-1 in tumour tissue was associated with reduced lymphangiosis carcinomatosa. In the tumourAng1-high group, 27 (67.5%) of the 40 patients had no presence of lymphangiosis carcinomatosa. In the tumourAng1-low group, 22 (45.8%) of the 48 patients had no lymphangiosis carcinomatosa (p = 0.042). Furthermore, the high abundance of Ang-1 in the tumour-infiltrating front correlated with a better histological grading. A well-differentiated (G1) tumour was diagnosed in 22 (84.6%) of the 26 patients in the invasive front^Ang1-high^ group, while only 30 (65.2%) of the 46 patients in the invasive front^Ang1-low^ group revealed a G1 tumour status (p = 0.038).

Similarly to Ang-1, Ang-2 was also uniformly dense in all tumour areas, including the tumour-infiltrating front; however, Ang-2 distribution was not significantly associated with clinicopathological characteristics in iCC.

### TEM frequency is associated with serum levels of carbohydrate antigen 19-9 (CA19-9) in patients with iCC

**Figure 2:**
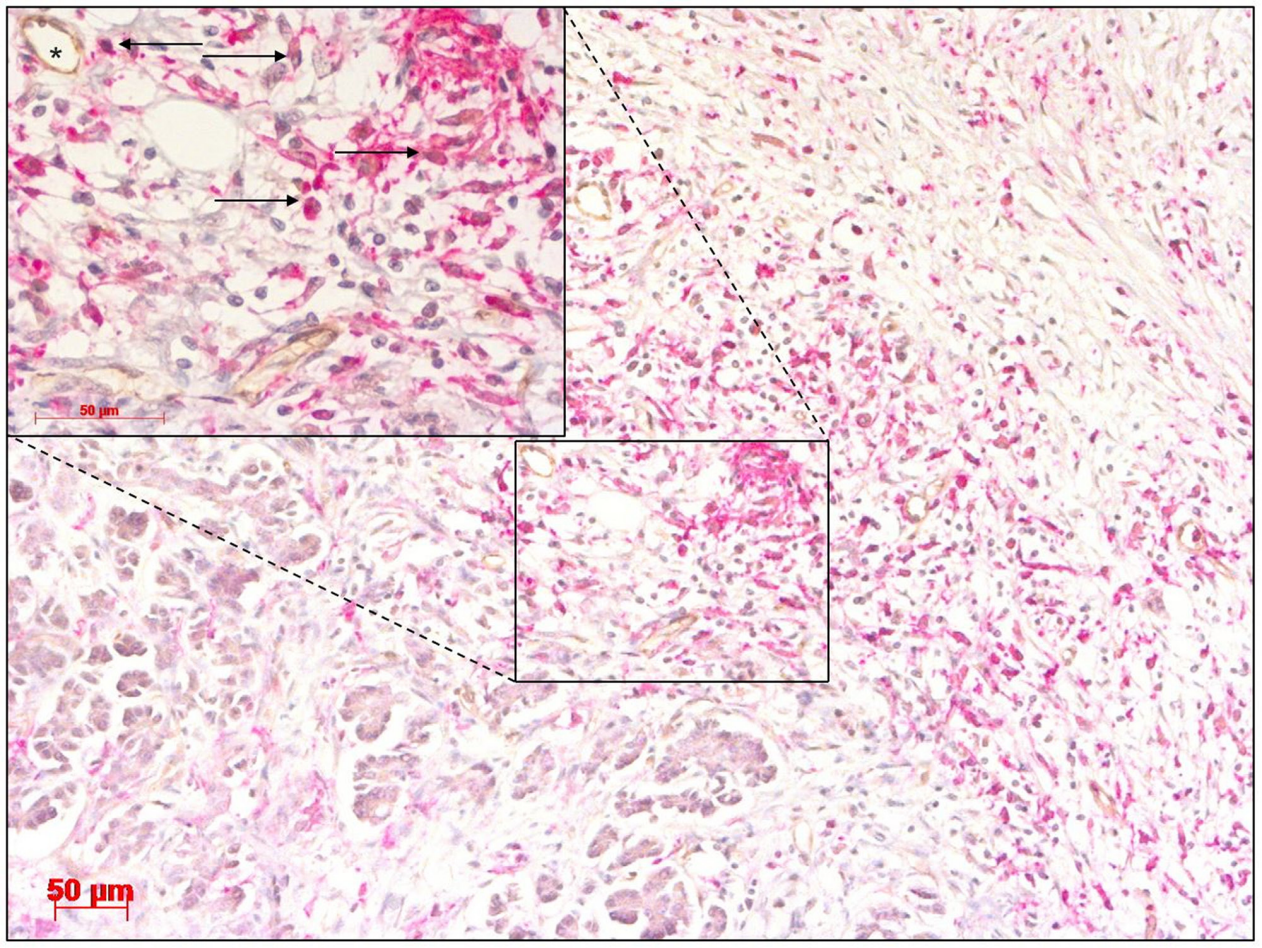
Cholangiocarcinoma stained with antibodies for CD14 and TIE2 showing a high abundance of TIE2-expressing monocytes (TEMs) (arrow) in close proximity to blood vessels (asterisk) Original magnification: x100. Detail of selected area, original magnification: x400. Scale bar 50 μm.

In addition to angiogenic miRNAs and the angiopoietin axis, this study also investigated the impact of TEMs in CCA. In iCC, TEMs displayed a uniform density in the tumour, tumour-infiltrating front and perivascular areas. The same expression pattern was previously described for HC (Figure 2) [31]. Interestingly, TEMs were observed in the tumour (tumour^TEM-positive^ group) and tumour-infiltrating front (invasive front^TEM-positive^ group) in 56 patients, while the rest of the tumour specimens (tumour^TEM-negative^ group, n = 33, and invasive front^TEM-negative^ group, n = 31) displayed a complete absence of infiltrating TEMs. No invading TEMs were observed in adjacent healthy liver tissue.

The absence of TEMs in the tumour-infiltrating front was associated with pathological CA19-9 levels. In the invasive front^TEM-positive^ group, 43 (79.6%) of the 54 patients had normal CA19-9 serum levels, whereas in the invasive front^TEM-negative^ group, only 15 (57.7%) of the 26 patients had normal values (p = 0.040).

The TEM density was not significantly associated with other clinicopathological markers or angiopoietin expression in iCC; however, a trend was observed between reduced local and overall tumour recurrence and mitigated formation of fibrosis in tumours with a high TEM density (data not shown).

### The influence of miRNAs and TEMs on overall and recurrence-free survival in patients with CCA

**Figure 3:**
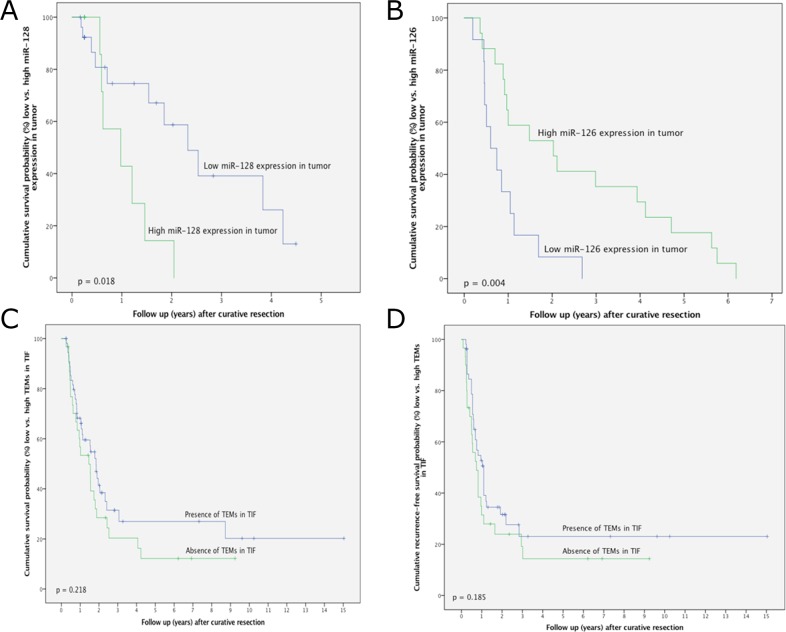
**(A)** Survival after intrahepatic cholangiocarcinoma surgery in relation to miR-128 expression in tumorous tissue. **(B)** Survival after hilar cholangiocarcinoma surgery in relation to miR-126 expression in tumorous tissue. **(C)** Survival after intrahepatic cholangiocarcinoma surgery in relation to the presence or absence of TEMs in the tumour-infiltrating front. **(D)** Recurrence-free survival after intrahepatic cholangiocarcinoma surgery in relation to the presence or absence of TEMs in the tumour-infiltrating front.

In iCC, survival was significantly decreased in patients with high relative miR-128 expression in the tumour in comparison to those with low miR-128 expression (p = 0.018; Figure 3A). The overall survival rates at 1-year postsurgery were 75.2% and 42.3% for patients with low and high miR-128 tumour expression, respectively. In HC, survival was significantly better in patients with high relative miR-126 expression in the tumour in comparison to those with low miR-126 expression (Figure 3B). No survival for longer than 3 years after resection was observed in patients with high miR-128 or low miR-126 expression in the tumour. Kaplan–Meier analysis with regards to the down- or upregulation of miRNAs relative to adjacent normal tissue revealed no significant differences (data not shown).

In patients with iCC, distinct trends towards improved overall and recurrence-free survival after resection were noted in those containing TEMs; however, this did not reach statistical significance. The overall survival rates were 67.8%, 32.4% and 27.6% at 1-, 3- and 5-years postsurgery, respectively, in patients with TEMs in the tumour-infiltrating front. Conversely, the overall survival rates were 53.1%, 20.2% and 12.8% at 1-, 3- and 5-years postsurgery, respectively, in patients without TEMs in the tumour-infiltrating front (p = 0.218) (Figure 3C). The 1-, 3- and 5-year recurrence-free survival rates in patients with tumours containing TEMs were 53.1%, 22.8% and 22.8%, respectively, whereas the 1-, 3- and 5-year recurrence-free survival rates in patients with tumours without TEMs were 31.9%, 18.4% and 13.8%, respectively (p = 0.185; Figure 3D).

### Prognostic significance of miRNAs and TEMs in CCA

This study also analysed whether miRNAs, Angs and TEMs, in addition to other clinicopathological parameters, could predict the outcome after resection for CCA. Clinicopathological parameters reported previously in the same cohort of patients included histological differentiation, T status, overall and local tumour recurrence, multiple tumour nodules, tumour size, perineural sheet infiltration and lymph node involvement [37]. Using multivariate analysis, the presence of TEMs in tumorous tissue, miR-126 and miR-128 were all identified as independent prognostic factors for survival (p = 0.040, p = 0.013 and p = 0.004, respectively; Table 2). With regards to recurrence-free survival, the presence of TEMs in the tumour-infiltrating front proved to be an independent prognostic variable in the multivariate analysis (*P* = 0.047; Table 2).

**Table 2:** Multivariate analysis of prognostic factors in patients with cholangiocarcinoma

Multivariate analysis (Overall Survival)				
Variable	Category	Odds ratio	p	Confidence interval
Histologic Differentiation	well or moderate/poor	2.266	0.040	1.038-4.946
T Status	T2/T3	1.688	0.236	0.710-4.009
Overall Tumor Recurrence	negative/positive	0.011	0.0001	0.001-0.093
Local Tumor Recurrence	negative/positive	0.707	0.401	0.315-1.588
Multiple Tumor Nodules	negative/positive	0.135	0.010	0.029-0.623
Tumor size	negative/positive	0.006	0.001	0.000-0.137
TEMs in tumor	negative/positive	2.423	0.040	1.042-5.633
miR-128 expression	low/high	38.976	0.004	3.257-466.374
miR-126 expression	low/high	0.322	0.013	0.131-0.789
**Multivariate analysis (Recurrence-free Survival)**				
**Variable**	**Category**	**Odds ratio**	**p**	**Confidence interval**
Tumor Nodules	solitary/multiple	0.301	0.001	0.149-0.606
Perineural Sheet Infiltration	negative/positive	0.370	0.008	0.178-0.772
Histologic Differentiation	well or moderate/poor	2.123	0.047	1.010-4.460
Lymph Node Involvement	negative/positive	0.542	0.066	0.282-1.042
TEMs in tumor invasive front	high/low	2.721	0.047	1.014-7.302

Following clinicopathological parameters were reported previously in the same cohort of patients: histologic differentiation, T status, overall and local tumor recurrence, multiple tumor nodules, tumor size, perineural sheet infiltration and lymph node involvement [37].

## DISCUSSION

This study analysed the tissue density of infiltrating TEMs and Angs and the expression of related angiogenic miRNAs in tumour samples from patients who underwent resection for CCA. The main findings were five-fold: (1) a high Ang-1 density was associated with reduced lymphangiosis carcinomatosa and improved histological grading in iCC; (2) angiogenic TEMs and miRNAs were differentially expressed in iCC and HC; (3) associated with established tumour markers, tumour growth and lymph node involvement; (4) served as independent prognosticators and (5) impacted on survival rates.

In the current work we show that high miR-126 and low miR-128 expression levels were associated with beneficial effects in CCA. Moreover, in accordance with previously published results in HC, the results showed that Ang-1 and TEMs correlated with improved tumour profiles and, together with angiogenic miRNAs, affected patient outcomes in CCA [28]. These findings suggest that ANG1–TIE2 signalling is potentially regulated by epigenetic miRNAs and influences TEM invasion resulting in beneficial effects in CCA; however, further studies are needed to shed light on the possible mechanisms.

Little information is currently available regarding the impact of angiogenesis in human CCA. Angiogenic miRNAs that regulate potent (lymph) angiogenic factors were shown to impact tumour progression in various models of human disease. Experimental data suggest a potential role of VEGF and angiopoietin-related pathways in CCA progression [38]. In addition, novel experimental and clinical research demonstrates that the impact of monocytes/macrophages on tumour progression varies with regards to the tumour type and the extent of neovascularization [39]. In comparison to HCC where the formation of prominent tumour neovascularization is a major factor influencing tumour progression, the extent of tumour-related neovascularization in CCA is uncertain. Therefore the current work focused on the importance of angiogenic miRNAs involved in the Ang- and VEGF-dependent molecular pathways. The next step explored the impact of angiogenic TEMs that directly respond to vascular growth factors on CCA progression and patient outcome.

Recent data suggest that miRNAs play important roles in liver biology and diseases, although the clinical impact of miRNAs in CCA remains to be further elucidated. Downregulation of miR-126 induces carcinogenesis, and miR-126 acts as a tumour suppressor and is also crucially involved in Ang-1 signalling and vessel maturation [9, 40–44]. Furthermore, miR-126 is also a potent modulator of VEGF signalling via KLF-2-dependent mechanisms, especially in the context of liver inflammation and cirrhosis. This is of potential importance for the presented results, as CCA tumour progression is highly dependent upon chronic hepato-biliary inflammation and injury [45].

**Figure 4:**
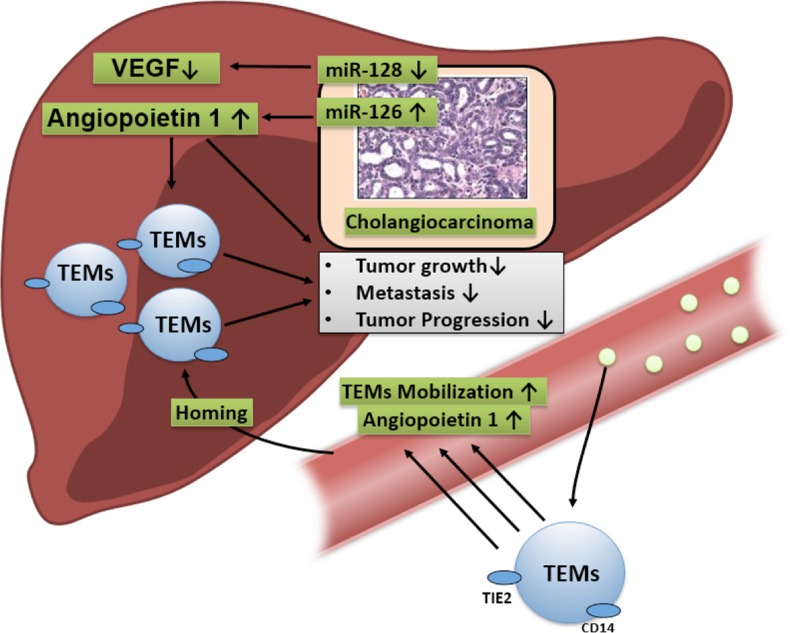
MiR-126 signalling is hypothesised to upregulate the Ang-1-dependent direct beneficial effects on tumour growth and indirect effects by fostering the homing TEMs in tumours, which in turn has a negative impact on CCA progression

Based on previous findings linking the angiopoietin axis to TEMs in HC, this study explored the impact of angiogenic miRNAs that influence Ang-1-related pathways on CCA progression. A potential mechanism was hypothesised that linked miR-126 to the activation of Ang-1-dependent mechanisms, which mediated enhanced infiltration of TEMs, which in turn exerted a beneficial effect on CCA progression (Figure 4). In this study, Ang-1 density correlated with reduced lymphangiosis carcinomatosa and improved histological grading. In addition, the multivariate analysis showed that Ang-1-regulating miR-126 and TEMs were associated with improved survival and prognosis. Thus TEMs seem to associate with prevailing beneficial miR-126 and Ang-1 profiles in CCA.

Given the growing interest in the miRNA-dependent modulation of (lymph) angiogenic signalling, the importance of other angiogenic miRNAs in CCA progression was investigated. Recent results are inconsistent; however, they do suggest that miR-128 is involved in malignant diseases, either as a tumour suppressor or oncogene [10, 46–54]. A tumour suppressive role of miR-128 was documented for glioma, neuroblastoma, prostate and nonsmall cell lung cancer, where abnormal miR-128 expression may impact the malignant phenotypes of cancer cells (i.e. proliferation, cell motility, invasion, apoptosis and self-renewal) [54, 55]. Conversely, in HCC and colorectal carcinoma, elevated serum and intratumoural levels of miR-128 were negatively associated with patient survival and disease prognosis [33].

This is the first work to document the expression of miR-128 in CCA. Moreover, it also shows that the upregulation of intratumoural miR-128 expression is a negative prognostic factor for patient survival following surgery (Table 1; Figure 3A). These data are consistent with studies that showed a negative prognostic value of elevated miR-128 serum levels in HCC patients [33]. However, most published scientific data showed that decreased miR-128 expression in tissue and serum samples was associated with tumour progression. This was linked to enhanced neovascularization via VEGF molecular pathways [55, 56]. The role of miR-128 in CCA progression is unknown. One possible explanation for these contradictory results is that in CCA dysregulation of miR-128 impacts tumour progression via mechanisms different from tumour related angiogenesis.

A negative prognostic value of the angiopoietin axis and TEMs was documented in highly vascularised malignancies; however, there is also a growing body of evidence that suggests that TEMs can have a positive impact. Research into HCC and pancreatic adenocarcinoma showed a negative influence of TEMs and Angs [40, 57–59]. Conversely, novel scientific data has linked TEMs to enhanced liver regeneration and a positive influence on patient outcome [60–62]. Moreover, Ang-1 and Ang-2 are functional antagonists, and a beneficial role of Ang-1 in human cancer has also been highlighted [17, 18].

The M1 or M2 polarisation state of TAMs differentially affects tumour progression and patient prognosis, therefore TIE2 expression on TEMs may be only the tip of the iceberg. In addition, further discrimination of distinct subpopulations within TEMs may deliver new insights regarding novel functions and differential influences on disease progression. It should be noted that results published to date that have revealed a negative prognostic value of TEMs have been performed in highly vascularised tumours where tumour progression relies on prominent tumour microvasculature [60, 63]. The extent of vascularisation in CCA is uncertain. In the current study, both Ang-1 and Ang-2 were not associated with the presence of TEMs; however, intratumoural Ang-1 expression was correlated with reduced lymphangiosis carcinomatosa and improved histological grading. These findings are in accordance with previous results that highlighted the beneficial effects of Ang-1 and TEMs in HC [28].

There is a scarce amount of data available with respect to the clinical significance of the angiopoietin axis and TEMs in CCA. Previously, the significance of TEMs was established with respect to their circulating blood population [39]. However, our group was the first to document the importance of TEM density in cancerous tissue and establish a link with the angiopoietin axis in HC [28]. Consistent with the findings in HC, the current work performed intratumoural visualisation of TEMs using immunoreactivity and revealed their preferential localisation in proximity to the microvasculature (Figure 2). Of note, a considerable number of tumours displayed a complete absence of TEMs.

The descriptive nature of the results is a limitation of the current work. Hence functional tests (i.e. *in vitro* co-culturing of TEMs and tumour-derived factors or tumour cells to measure and verify angiogenic responses will help to mechanistically elucidate the proposed effects. In summary, angiogenic miRNAs, the related Ang-1 axis and corresponding TEMs were associated with beneficial tumour profiles and improved outcomes in human CCA. However, further research is required to investigate the molecular mechanisms linking TEMs to miRNAs and related tumour angiogenesis.

## MATERIALS AND METHODS

### Patients and tumour samples

A total of 186 patients who underwent major hepatectomy for CCA (iCC or HC) at the Department of Surgery, Charité–Universitätsmedizin Berlin were included in the study. CCA was confirmed histopathologically and was classified according to the Union for International Cancer Control. The study was approved by the Ethics Committee of Charité–Universitätsmedizin Berlin (ID: 111 EA1/318/15).

Clinicopathological characteristics of the study population were reported previously [37]. Briefly, liver resection was in curative intent in all patients, and none of the patients received neoadjuvant radiotherapy or chemotherapy prior to surgery. None of the patients died in the postoperative period. Tissue blocks containing embedded representative samples of the tumours were retrieved from the archives at the Institute of Pathology. Histological diagnoses of the primary tumour stage and nodal status were determined from haematoxylin and eosin stained sections. Histological evaluation of all specimens was performed by an independent pathologist who had no knowledge of the prognosis or the clinicopathological variables. Cryopreserved (n = 88) and formalin-fixed paraffin-embedded (n = 88) tumour samples, which represented different patient collectives, were used. Analysis of miRNA expression was performed in the cryopreserved samples (iCC = 43; HC = 45), and Ang and TEM studies were performed in formalin-fixed tumour samples (iCC = 88).

### Immunohistochemistry

All protocols used for immunohistochemistry, histology, cellular infiltrate quantification and angiopoietin density were described in detail previously [28, 37, 64, 65].

### Quantification of TEM density

Briefly, TEMs were defined by their coexpression of CD14 and TIE2. The TEM density in the whole tumour area and in the tumour-infiltrating front was semiquantitatively scored as 1 = negative or 2 = positive. For statistical analysis, a score of 1 was categorised as the absence of abundance (or negative), while a score of 2 was categorised as the presence of abundance (or positive).

Patients with iCC were divided into groups either by the ‘positive’ or ‘negative’ abundance of TEMs in the tumour (tumour^TEM-positive^ group, n = 55, and tumour^TEM-negative^ group, n = 33) or by the ‘positive’ or ‘negative’ abundance of TEMs in the tumour-infiltrating front (invasive front^TEM-positive^ group, n = 56, and invasive front^TEM-negative^ group, n = 31). The tumour-infiltrating front was identified in 87 of the 88 tumour samples.

### Quantification of angiopoietin density

Briefly, Angs were defined by the expression of Ang-1 or Ang-2. The Ang density in the whole tumour area was semiquantitatively classified using the following categories: 0 = negative, 1 = 1%–25%, 2 = 26%–75% and 3 = >75%. For statistical analysis, scores of 0 and 1 were categorised as low expression, while scores of 2 and 3 were classified as high Ang expression. Ang density in the tumour-infiltrating front was semiquantitatively classified using the following categories: 0 = negative, 1 = 1%–25%, 2 = 26%–75% and 3 = >75%. For statistical analysis, scores of 0 and 1 were categorised as low abundance (or negative), while scores of 2 and 3 were categorised as high abundance (or positive).

Patients with iCC were divided into groups either by the ‘low’ or ‘high’ abundance of Angs in the tumour (tumour^Ang1-low^ group, n = 48; tumour^Ang2-low^ group, n = 66; tumour^Ang1-high^ group, n = 40 or tumour^Ang2-high^ group, n = 22) or by the ‘low’ or ‘high’ abundance of Angs in the tumour-infiltrating front (invasive front^Ang1-low^ group, n = 46; invasive front^Ang2-low^ group, n = 49; invasive front^Ang1-high^ group, n = 26 or invasive front^Ang2-high^ group, n = 23). The tumour-infiltrating front was identified in 72 of the 88 tumour samples stained for Ang-1 or Ang-2.

### Gene expression analysis

Samples of CCA and matched adjacent noncancerous liver tissue were collected after liver resection. Samples were snap frozen, and the relative expression of angiogenic miRNAs (hsa-miR-107 (MS00031255), which regulates VEGF-D signalling; hsa-miR-126 (MS00003430), which regulates Ang-1 signalling; hsa-miR-128 (MS00008582), which regulates VEGF-C signalling and hsa-miR-145 (MS00003528), which regulates Ang-2 signalling) was assessed by quantitative reverse transcriptase PCR [9, 32–34]. Total RNA (including miRNA) was isolated using Qiazol^®^ Lysis Reagent (Qiagen, Hilden, Germany) and purified as described in the user manual but with some modifications (miRNeasy System, Qiagen). Purified RNA was reverse transcribed, and qPCR was performed (miScript System II, Qiagen; RevertAid First Strand cDNA Synthesis Kit, Life Technologies, Karlsruhe, Germany and GoTaq qPCR Master Mix, Promega, Mannheim, Germany). RNU6 was used as an internal control in miRNA studies. The relative expression was calculated using the comparative ΔΔC_T_ method, and the values were expressed as 2^−ΔCT^ [66].

### Statistical analysis

Statistical analysis was performed using GraphPad Prism (Prism 6 for Macintosh Version 6.0b, GraphPad Software, La Jolla, CA) and SPSS software program (version 23). Data were described as means +/− standard deviations (SD) or standard error of mean (SEM, in figures). D'Agostino & Pearson omnibus normality test was used to test for normal distribution. For comparison of two groups unpaired *t*-test or non-parametric Mann-Whitney U test was used. A Two-Way Analysis of Variance (Two-Way ANOVA) was performed to test whether the two independent variables, entity’ (HC versus iCC) and ‘tissue sample’ (normal tissue versus tumor) affect the expression of the selected miRNAs. Two-Way ANOVA was followed by Sidak's multiple comparison test for a pairwise comparison. Survival analysis, univariate analysis and Kaplan–Meier curves were performed and generated using SPSS. Comparison of categorical and continuous variables was performed using the Chi-squared test and the Wilcoxon test, respectively. Survival data were compared using the log-rank test. Variables that significantly influenced survival in the univariate analysis were entered into a Cox regression analysis. When p ≤ 0.05 the differences were considered statistically significant.

## Abbreviations

Ang: angiopoietin
CCA: cholangiocarcinoma
HCC: hepatocellular carcinoma
HC: hilar cholangiocarcinoma
ICC: intrahepatic cholangiocarcinoma
TAMs: tumour-associated macrophages
TEMs: TIE2-expressing monocytes
VEGF: vascular endothelial growth factor
